# From Quiet Asthma to Clinical Remission: Can Optimized Maximal Inhaled Therapy Achieve Biologic-Free Remission in Difficult-to-Treat and Severe Asthma?

**DOI:** 10.3390/jcm15197757

**Published:** 2026-10-08

**Authors:** Vitaliano Nicola Quaranta, Silvano Dragonieri, Gianmarco Manzetti, Alessandra Tomasello, Alida Benfante, Nicola Scichilone, Paola Rogliani, Giovanna Elisiana Carpagnano

**Affiliations:** 1 Department of Translational Biomedicine and Neurosciences, Institute of Respiratory Disease, University of Bari “Aldo Moro”, 70124 Bari, Italy; 2 Unit of Respiratory Medicine, Department of Experimental Medicine, University of Rome “Tor Vergata”, 00133 Rome, Italy; 3 Division of Respiratory Medicine, PROMISE Department, Paolo Giaccone University Hospital, University of Palermo, 90127 Palermo, Italy; alessandra.tomasello01@unipa.it (A.T.); alida.benfante@unipa.it (A.B.);

**Keywords:** severe asthma, clinical remission, biologic-free remission, triple therapy, small-airway dysfunction, quiet asthma, inhaled therapy, difficult-to-treat asthma

## Abstract

Clinical remission, defined as sustained multidomain disease stability encompassing symptom control, freedom from exacerbations and from maintenance oral corticosteroid use, and stable or improved lung function, has emerged as a major therapeutic goal in severe asthma, largely driven by the success of biologic therapies. Consequently, remission is often regarded as an outcome primarily achievable through biologic treatment, whereas the potential contribution of optimized inhaled triple therapy has received comparatively less attention. This narrative review examines the evidence supporting biologic-free clinical remission across difficult-to-treat and severe asthma populations and explores the mechanisms that may contribute to sustained multidomain disease stability during optimized inhaled triple therapy. Importantly, we distinguish remission achieved after correction of previously unrecognized modifiable factors—which may reflect successful management of difficult-to-treat asthma—from on-treatment remission occurring in patients in whom severe asthma had already been established after appropriate treatment optimization. We review contemporary remission definitions, the biological rationale for remission achieved during optimized inhaled therapy, and evidence from historical cohorts, national registries, randomized clinical trials, post hoc analyses, and prospective real-world studies. Collectively, these data indicate that optimized inhaled triple therapy can be associated with clinically meaningful multidomain disease-stability states and, when established remission criteria are fulfilled, clinical remission, although the strength of evidence specifically in patients fulfilling current severe-asthma criteria remains more limited. Particular emphasis is placed on the contribution of small-airway dysfunction, the evolution of the quiet asthma concept as a proposed small-airway remission phenotype, and the potential roles of pharmacological synergy, improved peripheral drug deposition, and restoration of distal airway homeostasis. We also discuss the durability of remission, the need for continued multidomain follow-up, and the clinical and health-economic implications of achieving remission without biologic therapy. Current evidence suggests that optimized inhaled triple therapy should be considered not only a key component of severe asthma management but also a potential remission-oriented strategy in appropriately selected patients, including those who may achieve remission without subsequent biologic escalation. Future research should identify the phenotypes most likely to achieve biologic-free remission, clarify the underlying mechanisms, and prospectively validate remission-oriented inhaled treatment strategies.

## 1. Introduction

Severe asthma is increasingly framed not only as a disease to be controlled, but as a condition in which remission represents a realistic long-term therapeutic goal. This shift reflects a broader evolution in the management of chronic inflammatory diseases, where therapeutic success extends beyond symptom relief to sustained suppression of disease activity, prevention of future risk, minimization of treatment burden, and preservation of organ function. Accordingly, clinical remission, complete remission, and related low-disease-activity states have emerged as outcomes that more closely reflect the aspirations of both patients and clinicians than conventional measures of disease control [1,2,3,4,5].

Within this evolving therapeutic framework, it is essential to distinguish severe asthma from other forms of inadequately controlled disease. Asthma that remains inadequately controlled should first be distinguished from truly severe disease. According to the Global Initiative for Asthma (GINA), uncontrolled asthma is characterized by poor symptom control and/or frequent exacerbations requiring oral corticosteroids or serious exacerbations requiring hospitalization. Difficult-to-treat asthma refers to asthma that remains uncontrolled despite prescription of medium- or high-dose inhaled corticosteroid (ICS) therapy combined with a second controller, usually a long-acting β_2_-agonist (LABA), or that requires high-dose treatment to maintain adequate control. Severe asthma represents a subset of difficult-to-treat asthma and is defined as asthma that remains uncontrolled despite good adherence to maximally optimized high-dose ICS/LABA therapy and appropriate management of contributory factors, or that worsens when high-dose treatment is reduced [6]. Importantly, asthma should not be classified as severe when control substantially improves after correction of modifiable factors such as poor adherence, incorrect inhaler technique, relevant exposures, or untreated comorbidities [6].

This distinction is also epidemiologically relevant. Severe asthma represents only a minority of the overall asthma population. GINA reports that approximately 24% of adults with asthma receive Step 4–5 treatment and around 17% may have difficult-to-treat asthma, whereas approximately 3.7% fulfil criteria for severe asthma after accounting for symptom control, adherence, and inhaler technique. These estimates underline the importance of distinguishing true severe asthma from uncontrolled disease driven by potentially modifiable factors [6]. This distinction is particularly relevant when interpreting remission after treatment optimization. If disease control is achieved primarily through correction of poor adherence, inhaler misuse, untreated comorbidities, or other modifiable factors, the patient may more appropriately be considered to have difficult-to-treat rather than severe asthma. Conversely, current GINA criteria do not require previous exposure to a LAMA or to a single-inhaler triple combination before severe asthma can be established or biologic therapy considered. Therefore, a patient with confirmed severe asthma despite optimized high-dose ICS/LABA therapy may subsequently achieve on-treatment remission after further optimization with ICS/LABA/LAMA without invalidating the original severe-asthma classification [6].

The growing interest in remission has largely paralleled the development of biologic therapies, which have transformed the management of severe type 2 asthma and established remission as a measurable therapeutic objective [3,4,5]. Consequently, remission is often perceived as a biologic-era phenomenon. However, many patients undergo prolonged periods of optimized inhaled therapy before biologic initiation, during which careful correction of modifiable factors and treatment optimization may lead to sustained multidomain improvement and, in selected cases, clinical remission. Despite its potential clinical relevance, this therapeutic pathway has received comparatively little systematic attention because most remission evidence derives from biologic-treated cohorts, whereas data supporting remission with inhaled therapy alone remain fragmented across natural-history studies, optimization studies, and more recent real-world and randomized investigations [2,3,4,5,6,7,8,9,10]. This interpretation is consistent with the emerging disease-modification framework, in which remission is increasingly considered a treatment goal across the asthma severity spectrum and may occur on treatment with either inhaled disease-modifying therapy or biologic agents [11].

Against this background, this review synthesizes the available evidence supporting biologic-free clinical remission achieved with optimized maximal inhaled therapy across difficult-to-treat and severe asthma populations. We first examine current definitions of clinical remission and the biological rationale for remission achieved during optimized inhaled therapy, before reviewing the historical and contemporary clinical evidence. We then discuss the mechanistic contribution of optimized inhaled triple therapy, defined as the combination of an inhaled corticosteroid, a long-acting β_2_-agonist, and a long-acting muscarinic antagonist (ICS/LABA/LAMA), the evolution of the quiet asthma concept as a proposed small-airway phenotype and conceptual model, the durability of remission and risk of relapse, and finally the clinical, economic, and research implications of this emerging therapeutic paradigm.

### Search Strategy and Review Methodology

This narrative review was guided by the following research question: can optimized maximal inhaled therapy, particularly ICS/LABA/LAMA therapy, be associated with sustained clinical remission or remission-like states in patients with moderate-to-severe or severe asthma without biologic treatment, and which clinical, physiological, and pharmacological factors may contribute to this outcome?

A literature search was conducted using PubMed/MEDLINE, Scopus, and Web of Science, supplemented by screening the reference lists of relevant original articles, reviews, consensus documents, and international asthma guidelines. The search included publications available from database inception to September 2026. All authors contributed to the literature search, identification of potentially relevant publications, and selection of evidence included in the narrative synthesis.

Search terms were combined using the Boolean operators AND and OR and included combinations of the following terms: asthma, severe asthma, moderate-to-severe asthma, clinical remission, asthma remission, biologic-free remission, remission without biologics, inhaled therapy, triple therapy, ICS/LABA/LAMA, single-inhaler triple therapy, small airways, small airway dysfunction, quiet asthma, disease control, and oral corticosteroids. Additional targeted searches were performed to identify studies evaluating treatment optimization, small-airway physiology, triple inhaled therapy, and individual domains contributing to contemporary definitions of clinical remission.

Given the limited number of studies specifically addressing clinical remission without biologic therapy, a broad narrative approach was adopted. Eligible publications included prospective and retrospective observational studies, national registry studies, randomized controlled trials, post hoc analyses of randomized trials, prospective real-world studies, longitudinal cohorts, relevant systematic and narrative reviews, consensus documents, and international guidelines. Studies were considered when they directly evaluated clinical remission or provided relevant evidence regarding key remission domains, including symptom control, exacerbation freedom, oral corticosteroid exposure, and lung function.

Studies focusing exclusively on biologic-treated populations were not considered primary evidence of biologic-free remission, although selected studies were included when they contributed to definitions of remission, predictors of remission, or the overall conceptual framework. Pediatric-only studies, isolated case reports, and publications not directly relevant to asthma remission, treatment optimization, or the mechanistic basis of disease stability achieved during optimized inhaled therapy were excluded. Because of substantial heterogeneity in study populations, treatment regimens, remission definitions, and study designs, no formal meta-analysis or quantitative evidence synthesis was performed.

The selected literature was organized into four main domains: (1) definitions and conceptual frameworks of asthma remission; (2) historical evidence of spontaneous or treatment-free remission; (3) contemporary evidence of remission during optimized inhaled therapy, including randomized trials, post hoc analyses, registries, and real-world studies; and (4) mechanistic evidence addressing triple therapy, small-airway dysfunction, extrafine drug deposition, and the quiet asthma phenotype.

To improve the transparency and quality of the narrative synthesis, the preparation and revision of this review were guided by the Scale for the Assessment of Narrative Review Articles (SANRA) framework. Accordingly, particular attention was given to the justification of the review’s relevance, formulation of a focused research question, description of the literature search, appropriate referencing, critical appraisal and contextualization of the different levels and types of evidence, and clear presentation of clinically relevant endpoint data [12].

## 2. Defining Clinical Remission in Difficult-to-Treat and Severe Asthma

For clarity, the terminology used throughout this review is defined as follows. “Clinical remission” is used as the overarching term for sustained multidomain disease stability, generally encompassing absence of exacerbations and maintenance oral corticosteroid use, good symptom control, and stable or improved lung function. “Complete clinical remission” and “partial clinical remission” are used specifically according to the Severe Asthma Network Italy (SANI) Delphi framework, in which complete clinical remission requires fulfillment of all four clinical domains, whereas partial clinical remission requires absence of oral corticosteroid use together with at least two of the remaining three domains. The term “complete remission” is reserved for more stringent concepts that may additionally require normalization of underlying disease activity, including airway hyperresponsiveness and/or inflammatory biomarkers. Finally, “remission-like state” is used descriptively when substantial multidomain disease stability is demonstrated but a formally established remission definition is not applied or fully satisfied [1,2,6]. For the purposes of this review, “clinical remission” is the primary operative term and refers to sustained multidomain disease stability characterized by absence of exacerbations and maintenance oral corticosteroid use, good symptom control, and stable or improved lung function; where individual studies apply more specific definitions, such as complete or partial clinical remission, these are reported using the terminology of the original study.

The modern conceptual basis for remission in asthma was established through an expert consensus framework that defined remission as a multidomain construct, extending beyond symptom control alone to encompass sustained disease stability. Clinical remission was proposed as a state sustained for at least 12 months and characterized by absence of significant symptoms according to a validated instrument, optimization or stabilization of lung function, patient–healthcare provider agreement regarding remission, and no systemic corticosteroid use [1]. Complete remission additionally requires objective resolution of asthma-related inflammation and, where appropriate, negative bronchial hyperresponsiveness, whereas remission off treatment requires no asthma treatment for at least 12 months [1].

This framework also distinguished clinical remission from complete remission, thereby recognizing that not all patients achieving sustained clinical stability necessarily demonstrate complete resolution of the underlying disease process [2]. This distinction is essential when evaluating remission without biologics, because many patients managed successfully with maximal inhaled therapy may meet clinical, but not complete, remission criteria.

Among severe-asthma-specific definitions, the Severe Asthma Network Italy (SANI) Delphi consensus provides a practical multidomain framework for defining clinical remission. According to this consensus, complete clinical remission requires the simultaneous fulfillment of four criteria: absence of oral corticosteroid use, absence of asthma symptoms, absence of exacerbations or attacks, and stability of pulmonary function. Partial clinical remission is defined by the absence of oral corticosteroid use together with fulfillment of at least two of the remaining three criteria: absence of symptoms, absence of exacerbations or attacks, and stable pulmonary function [2]. This graded approach recognizes that clinically meaningful improvement may occur even when all domains required for complete clinical remission are not simultaneously achieved.

These criteria are broadly aligned with the conceptual framework presented in the GINA 2026 Strategy Report and Severe Asthma Guide. GINA recognizes clinical remission on treatment as a relevant therapeutic outcome and notes that proposed definitions generally incorporate sustained absence of asthma symptoms and exacerbations, no oral corticosteroid use, and stable or improving lung function over a defined period [6]. However, GINA does not currently endorse a single universal operational definition of remission. It also emphasizes that clinical remission should not be equated with cure, since airway hyperresponsiveness and/or inflammation may persist despite apparent clinical quiescence. More stringent definitions of complete remission may therefore additionally require normalization of airway responsiveness and/or inflammatory markers [6]. In this context, the SANI criteria provide a pragmatic severe-asthma-specific framework that closely reflects the principal clinical domains highlighted by GINA while allowing complete and partial remission to be distinguished in clinical practice and research.

Later reviews and commentaries have reinforced these principles, positioning clinical remission as a therapeutic target that integrates current disease status with future risk, rather than focusing solely on short-term symptom control [3,4,5]. This concept has important implications for inhaled-only treatment strategies. Apparent symptom improvement alone is insufficient. A patient who feels better on maximal inhaled therapy but continues to exacerbate, remains dependent on maintenance oral corticosteroids, or loses lung function should not be considered in remission [1,2,3,4,5,6].

## 3. Why Maximal Inhaled Therapy May Support Remission

Current GINA recommendations emphasize optimization of high-dose ICS-containing therapy and systematic assessment of potentially modifiable factors before confirming a diagnosis of severe asthma and proceeding to biologic treatment. In this context, high-dose inhaled therapy generally refers to treatment with a high-dose inhaled corticosteroid in combination with a long-acting β_2_-agonist (ICS/LABA), with addition of a long-acting muscarinic antagonist (LAMA) when clinically appropriate as part of maximal inhaled treatment. Importantly, treatment optimization should also include verification and correction of inhaler technique and adherence, assessment of relevant comorbidities such as obesity, chronic rhinosinusitis, gastroesophageal reflux disease, obstructive sleep apnea, inducible laryngeal obstruction, bronchiectasis, COPD, anxiety and depression, and identification of modifiable exposures or triggers including smoking, vaping, environmental or occupational exposures, allergens in sensitized patients, air pollution, and medications that may worsen asthma, such as beta-blockers or non-steroidal anti-inflammatory drugs. Excessive reliance on short-acting β_2_-agonists, medication-related adverse effects, and relevant social or economic barriers to treatment should also be considered [6]. Throughout this review, ‘optimized inhaled therapy’ refers broadly to appropriate inhaled treatment after correction of adherence, inhaler technique, and other modifiable factors; ‘maximal inhaled therapy’ refers to optimized high-dose ICS/LABA with addition of LAMA when clinically appropriate; and ‘triple therapy’ specifically denotes ICS/LABA/LAMA [6]. The term ‘extrafine triple therapy’ is reserved for formulations designed to enhance peripheral lung deposition, and the proposed small-airway mechanistic advantages discussed below should therefore not be extrapolated to all triple-therapy formulations [13].

Beyond optimization of inhaled therapy and correction of modifiable factors, additional non-biologic add-on treatments may be considered in selected patients with persistent uncontrolled asthma. In accordance with current GINA recommendations, leukotriene receptor antagonists (LTRAs) represent an additional controller option, particularly in patients with relevant associated features such as allergic rhinitis or aspirin-exacerbated respiratory disease. Low-dose azithromycin may also be considered in adults with persistent uncontrolled asthma despite optimized high-dose ICS/LABA treatment, following specialist assessment and appropriate safety evaluation. Before initiating long-term azithromycin, potential risks including QTc prolongation, gastrointestinal adverse effects, non-tuberculous mycobacterial infection, and antimicrobial resistance should be considered. These therapies may therefore form part of a comprehensive non-biologic optimization strategy before or alongside assessment for biologic treatment, although they should not be regarded as mandatory prerequisites when biologic therapy is otherwise clinically indicated [6].

Although remission following correction of previously unrecognized modifiable factors may identify patients with difficult-to-treat rather than truly severe asthma, this distinction does not apply to all responders to optimized triple therapy. Because GINA does not require prior LAMA treatment or use of a single-inhaler triple combination to establish severe asthma, patients with appropriately confirmed severe asthma on optimized high-dose ICS/LABA may subsequently achieve on-treatment remission after escalation or optimization to ICS/LABA/LAMA [6].

This possibility is supported by a plausible pathophysiological rationale. High-dose inhaled corticosteroids suppress airway inflammation; long-acting beta_2_-agonists improve bronchodilation and symptom stability; long-acting muscarinic antagonists enhance bronchodilation and may reduce exacerbation burden in selected patients; and extrafine formulations increase drug deposition in the distal lung, where small-airway dysfunction may contribute substantially to persistent symptoms and poor control [6,13,14,15,16,17,18]. These complementary mechanisms target multiple components of asthma pathophysiology simultaneously, providing a biological basis for sustained disease stabilization. This rationale is further supported by experimental evidence showing that indacaterol, glycopyrronium, and mometasone can interact pharmacologically to enhance bronchorelaxation and attenuate inflammatory responses in hyperresponsive airways, supporting the concept that ICS/LABA/LAMA combinations may exert integrated effects beyond the simple addition of their individual components [19]. In parallel, contemporary evidence on extrafine fixed-dose triple combinations supports the clinical relevance of this pharmacological framework across both randomized trials and real-world settings. Enhanced distal-airway drug delivery may be particularly relevant in patients with evidence of small-airway dysfunction, including persistent peripheral airflow limitation, abnormal oscillometric indices, reduced mid-expiratory flows, air trapping or hyperinflation, and in patients with persistent symptoms or exacerbations despite otherwise optimized inhaled treatment. In these clinical settings, targeting the peripheral airways may be especially relevant because residual distal-airway inflammation and dysfunction can persist despite apparently adequate control of the larger airways [13,15,20]. Thus, the clinical benefit of triple therapy should primarily be interpreted in relation to the combined pharmacological effects of ICS, LABA, and LAMA. Enhanced peripheral deposition with extrafine formulations may provide an additional theoretical advantage in patients with prominent small-airway dysfunction, but its independent contribution to clinical remission has not been established. Importantly, remission has also been reported with non-extrafine FF/UMEC/VI, indicating that remission cannot currently be attributed specifically to particle size or distal deposition [6,13,14,15,16,17,18,19,20].

## 4. Evidence for Remission Without Biologic Therapy

### 4.1. Historical Perspective

The literature specifically addressing remission in severe asthma without biologics is limited, making historical natural-history cohorts an important source of indirect evidence. One of the most informative is the northern European arm of the European Community Respiratory Health Survey. In that prospective cohort, remission was defined as the absence of asthma symptoms for two consecutive years together with no current asthma medication. The average remission rate was 20.2 per 1000 person-years, indicating that treatment-free remission is biologically achievable, although relatively uncommon. Remission was more likely in individuals who stopped smoking and in those with milder disease at baseline, whereas persistent symptoms reduced the likelihood of remission. Taken together, these findings indicate that remission without biologic therapy is possible but strongly influenced by baseline disease severity and chronicity [7].

Long-term observational evidence further reinforces this concept. In the 30-year Danish adult asthma cohort, clinical remission was defined as no asthma symptoms and no prescribed asthma medication during the previous year, whereas complete remission additionally required low exhaled nitric oxide, absence of bronchodilator reversibility, absence of airway hyperresponsiveness, and absence of airflow limitation. After approximately three decades of follow-up, 25% of participants met criteria for clinical remission and 15% for complete remission. However, longer symptom duration before specialist assessment reduced the probability of remission, and greater baseline severity predicted poorer long-term outcomes. These observations indicate that the likelihood of remission progressively declines as asthma becomes more longstanding and structurally established, a finding that is particularly relevant to patients with severe disease [8].

A broader review of asthma remission published in 2022 reached similar conclusions. Across adult studies, reported remission rates varied widely because definitions and populations were heterogeneous, but spontaneous or treatment-free remission was consistently less common among patients with more severe and long-standing disease. Importantly, the review identified a major gap in the available evidence: most historical remission studies investigated spontaneous remission rather than remission induced by optimized conventional therapy, and almost none specifically evaluated patients treated at contemporary GINA step 4 or 5 intensity [21]. Consequently, although the historical literature demonstrates that remission without biologic therapy is biologically possible, it provides only indirect evidence regarding whether clinical remission can be achieved during optimized contemporary inhaled therapy in patients with true severe asthma. This gap has begun to be addressed only by more recent registry analyses, randomized trials, and real-world studies evaluating remission under maximal inhaled therapy. Accordingly, these historical cohorts are considered here primarily as evidence of the biological possibility of spontaneous or treatment-free asthma remission and should not be interpreted as evidence that contemporary optimized inhaled therapy itself caused remission; the potential contribution of inhaled treatment is therefore evaluated separately using contemporary interventional and real-world evidence.

### 4.2. Contemporary Evidence

Although dedicated prospective studies specifically evaluating remission with inhaled therapy alone remain limited, recent evidence increasingly supports the concept that optimized maximal inhaled therapy can be associated with clinically meaningful multidomain disease stability and, where prespecified remission criteria are fulfilled, clinical remission. Interpretation of these studies requires attention to baseline treatment optimization and disease classification: some cohorts include patients with moderate-to-severe or difficult-to-treat asthma, whereas others include patients already classified within severe-asthma settings. Accordingly, remission following inhaled therapy should be interpreted in relation to the treatment status and severity definition used in each individual study.

A major contribution comes from the SANI registry, in which patients receiving ICS/LABA/LAMA without biologics were evaluated using multidomain clinical remission criteria. After 12 months, 34.5% achieved complete clinical remission and 34.5% partial clinical remission, providing the first large real-world evidence that remission is achievable in a substantial proportion of patients managed with inhaled therapy alone [9]. Although this cohort probably represented a less severe population than patients requiring biologics, these findings challenged the view of inhaled therapy as only an intermediate step before biologic treatment and supported its potential independent role as a remission-oriented strategy [9].

Importantly, these findings should be interpreted in the context of the substantial efficacy of biologic therapies in severe asthma. In the same SANI analysis, complete clinical remission at 12 months was achieved by 45.8% of biologic-treated patients compared with 34.5% of patients receiving inhaled therapy without biologics [9]. These data reinforce that biologics remain highly effective remission-oriented therapies in eligible patients with severe asthma. However, available comparisons between biologic and inhaled-treatment cohorts are largely non-randomized and involve populations with different baseline disease characteristics; therefore, they should not be interpreted as direct head-to-head evidence of therapeutic superiority. Rather, the emerging evidence for biologic-free remission identifies a complementary therapeutic opportunity in appropriately selected patients whose disease remains responsive to optimized inhaled therapy.

This concept has been further reinforced by evidence showing that optimization of background inhaled therapy actively influences the likelihood of achieving clinical remission, highlighting its independent contribution to disease stabilization rather than simply accompanying biologic treatment [22]. Consistent with this view, remission in the SANI analysis was associated with baseline clinical characteristics such as asthma control, preserved lung function, and treatable traits including small-airway dysfunction, whereas conventional type 2 biomarkers showed little predictive value, suggesting that remission achieved with inhaled therapy alone may depend more on clinical phenotype than on inflammatory biomarkers [2,4,9,17,23]. Contemporary reviews have similarly emphasized that optimization of inhaled therapy remains a central component of the pathway toward both disease control and clinical remission [3,10].

Evidence from randomized trials and prospective studies has further strengthened this framework, although the populations studied should be distinguished carefully. CAPTAIN enrolled patients with inadequately controlled moderate-to-severe asthma despite ICS/LABA rather than a population specifically meeting current severe-asthma criteria; its post hoc analyses nevertheless demonstrated higher multidomain remission rates with FF/UMEC/VI than with FF/VI [24,25]. Similarly, in the prospective real-world TriMaximize study of 1445 patients with moderate-to-severe asthma treated with extrafine BDP/FOR/G, four-component clinical remission was achieved by 39.5% of patients in the first year and 47.9% in years two to three [26]. TRIMARAN and TRIGGER enrolled patients with uncontrolled asthma and at least one previous exacerbation despite medium- or high-dose ICS/LABA [27], while ATLANTIS included adults with asthma across a broad severity spectrum [28]. PERFORM evaluated a broad pragmatic population with uncontrolled, predominantly infrequently exacerbating asthma and therefore provides evidence for the remission potential of triple therapy in uncontrolled asthma, rather than specifically in established severe asthma [29]. By contrast, the SANI registry and prospective quiet-asthma cohorts provide evidence from patients managed within severe-asthma settings [9,15,16]. Taken together, these studies support a remission-oriented role for optimized inhaled therapy across the difficult-to-treat/severe-asthma treatment continuum, while the evidence specifically confined to rigorously defined severe asthma remains more limited. Notably, remission has been observed with both extrafine and non-extrafine triple-therapy regimens; therefore, the available clinical evidence does not permit attribution of remission specifically to particle size or enhanced peripheral deposition.

**Figure 1:**
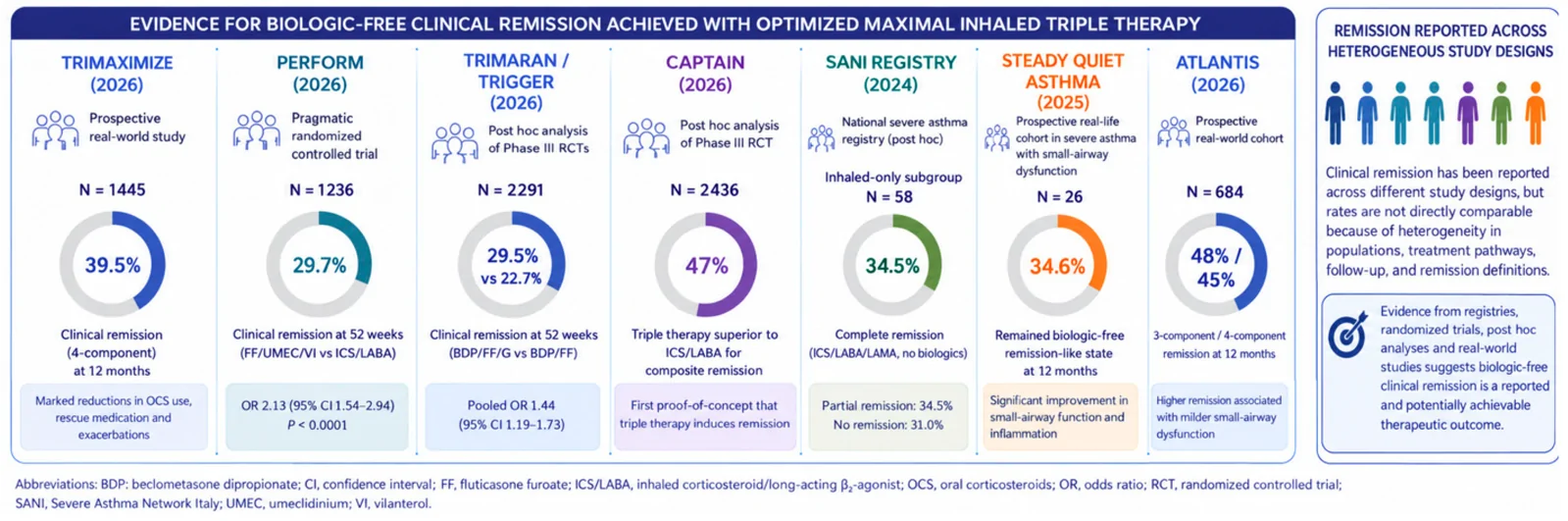
Evidence supporting biologic-free clinical remission with optimized inhaled triple therapy across uncontrolled, difficult-to-treat, and severe asthma populations. Remission criteria are shown in abbreviated, study-specific form. All composite endpoints required multidomain stability, typically including absence of severe exacerbations, no maintenance or frequent oral/systemic corticosteroid use, well-controlled symptoms (ACT or ACQ thresholds), and stable or improved lung function (FEV_1_). Exact definitions and thresholds are reported in the original publications. Disease severity and the degree of prior inhaled-treatment optimization differed across studies; therefore, remission outcomes should be interpreted according to the baseline population and treatment pathway of each study. Abbreviations: ICS = inhaled corticosteroid; LABA = long-acting β_2_-agonist; LAMA = long-acting muscarinic antagonist; BDP = beclometasone dipropionate; FOR = formoterol; FF = fluticasone furoate; G = glycopyrronium; FF/UMEC/VI = fluticasone furoate/umeclidinium/vilanterol; SCS/OCS = systemic/oral corticosteroids; ACT = Asthma Control Test; ACQ = Asthma Control Questionnaire; FEV_1_ = forced expiratory volume in 1 s; RCT = randomized controlled trial; SANI = Severe Asthma Network Italy.

**Figure 2:**
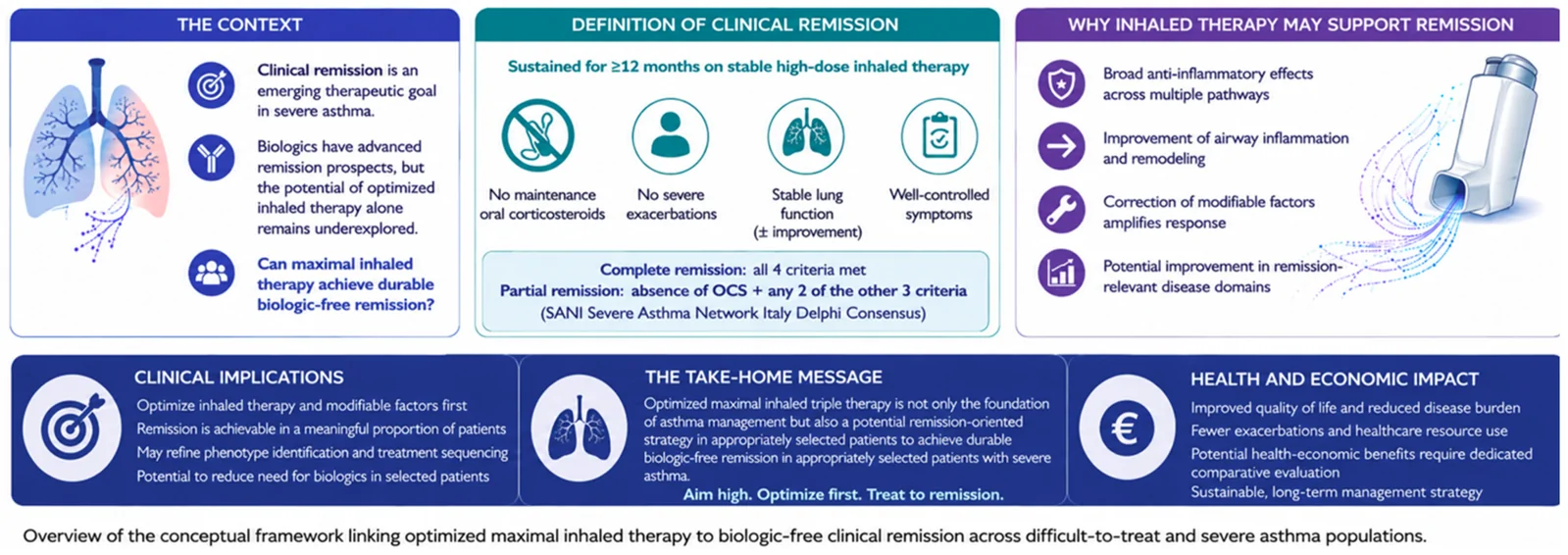
Conceptual framework of biologic-free clinical remission with optimized maximal inhaled therapy across the difficult-to-treat and severe asthma treatment pathway. Overview of randomized trials, real-world studies, post hoc analyses, and registry data evaluating biologic-free clinical remission across populations differing in baseline asthma severity and degree of prior treatment optimization. Across different study designs, clinically relevant remission rates have been reported, although direct comparison is limited by heterogeneity in asthma severity, prior treatment optimization, and remission definitions. Disease severity and the degree of prior inhaled-treatment optimization differed across studies; therefore, remission outcomes should be interpreted according to the baseline population and treatment pathway of each study. Abbreviations: ACT, Asthma Control Test; ACQ, Asthma Control Questionnaire; BDP, beclometasone dipropionate; CI, confidence interval; FF, fluticasone furoate; FEV_1_, forced expiratory volume in 1 s; G, glycopyrronium; ICS, inhaled corticosteroid; LABA, long-acting β_2_-agonist; LAMA, long-acting muscarinic antagonist; OCS, oral corticosteroids; OR, odds ratio; RCT, randomized controlled trial; SANI, Severe Asthma Network Italy; UMEC, umeclidinium; VI, vilanterol.

Collectively, evidence from registries, randomized trials, post hoc analyses, and real-world studies indicates that optimized inhaled triple therapy can represent a remission-oriented strategy across appropriately selected uncontrolled, difficult-to-treat, and severe asthma populations. However, because several major studies were not conducted specifically in patients fulfilling current severe-asthma criteria, reported remission rates should be interpreted according to the baseline asthma population, treatment intensity, and remission definition used in each study [24,25,26,27,28,29] (Table 1; Figure 1 and Figure 2).

## 5. Triple Therapy as a Potential Bridge Between Disease Control and Remission

A plausible pharmacological rationale for the multidomain benefits observed with optimized inhaled triple therapy comes from the work of Rogliani and colleagues, who reported pharmacological interactions among ICS, LABA, and LAMA that may produce effects greater than the simple sum of their individual components. Experimental and preclinical studies have demonstrated enhanced bronchorelaxation and suggested complementary anti-inflammatory effects [14,19]. However, these findings should be regarded as mechanistic support for triple therapy rather than evidence that pharmacological synergy itself induces clinical remission, since this causal relationship has not been demonstrated in prospective clinical studies. These pharmacological effects relate to the addition of LAMA and triple therapy as a drug-class strategy and should be distinguished from any additional effect potentially related to aerosol particle size or peripheral deposition.

These complementary pharmacological actions provide a biologically plausible explanation for improvements across several domains that contribute to contemporary remission definitions, including symptom control, exacerbation prevention, and lung function. Nevertheless, whether the proposed pharmacological synergy specifically contributes to the transition from disease control to sustained clinical remission remains uncertain and should be considered hypothesis-generating rather than established.

Clinical evidence demonstrates the efficacy of triple therapy across several individual domains that are also components of remission definitions. Network meta-analyses have shown that ICS/LABA/LAMA improves lung function and reduces moderate-to-severe exacerbations compared with ICS/LABA, particularly with high-dose inhaled corticosteroids, while maintaining an acceptable safety profile [17]. Importantly, these analyses were not designed to evaluate clinical remission and therefore cannot establish that the observed physiological or clinical improvements translate directly into remission. Rather, they demonstrate that triple therapy favorably affects several of the component outcomes required for remission. Accordingly, physiological improvement, disease control, and clinical remission should be considered distinct levels of therapeutic response. Improvements in lung function, airway inflammation, or exacerbation burden may contribute to disease control, but clinical remission requires sustained simultaneous fulfilment of multidomain criteria and cannot be inferred from improvement in individual physiological outcomes alone.

Accordingly, optimized triple therapy may be considered a remission-oriented treatment strategy in appropriately selected patients, based on its demonstrated effects on multiple remission-relevant domains and the emerging clinical evidence of multidomain remission with inhaled therapy. However, current data do not establish that pharmacological synergy itself is the mechanism responsible for remission. When improvements in symptoms, exacerbations, corticosteroid exposure, and lung function are simultaneously achieved and sustained, patients may fulfil contemporary clinical remission criteria [1,2,3,4,5,17,18]. This concept is supported by severe asthma-specific remission definitions and emerging real-world evidence demonstrating that optimized inhaled therapy alone can achieve biologic-free remission in selected patients [2,9,15,16,17,18].

## 6. Quiet Asthma: A Proposed Small-Airway Remission Phenotype

Although the concept of clinical remission is now well established, the terminology used to describe sustained low disease activity remains less clearly defined. Among these expressions, *quiet asthma* has intuitive clinical appeal but has not been incorporated into current international remission frameworks. Historically, it has been used to describe clinical quiescence or low symptom burden, whereas terms such as *silent asthma* or *silent chest* refer to severe acute airflow limitation rather than remission. Accordingly, *quiet asthma* should currently be regarded as a descriptive concept rather than a validated clinical endpoint [1,2,3,4,5,13].

An important conceptual precursor is the ‘quiet zone’ concept described by Lipworth et al., which identifies the small airways—commonly defined as peripheral airways with an internal diameter <2 mm—as a frequently overlooked site of persistent inflammation and airflow limitation despite apparently adequate treatment. Small-airway dysfunction refers to functional and/or structural abnormalities affecting this peripheral compartment and may be identified using complementary physiological measures, including oscillometry, mid-expiratory flows, lung-volume assessment, and other techniques, as no single test currently represents a definitive gold standard [13,30]. This concept highlighted the potential contribution of distal airway dysfunction to poor asthma control and provided the rationale for targeting the peripheral lung with optimized inhaled therapy. Although the concept of “quiet asthma” was conceptually inspired by the growing recognition of the small airways as a clinically relevant compartment, it should not be regarded as a direct extension or validated evolution of the established “quiet zone” concept. “Quiet asthma” is a recently proposed, hypothesis-generating construct developed primarily from studies by our group, describing sustained clinical and small-airway stability during optimized inhaled therapy. Accordingly, it requires confirmation in independent cohorts before it can be considered a validated phenotype or incorporated into remission frameworks [15,16].

Building on this framework, optimized extrafine single-inhaler triple therapy was shown to improve asthma control, airway inflammation, and physiological markers of small-airway function in patients with severe asthma and small-airway dysfunction, leading our group to propose the descriptive concept of ‘quiet asthma’ [15]. A plausible pharmacological rationale for the observations underlying this proposed phenotype includes the complementary bronchodilator and anti-inflammatory effects of ICS/LABA/LAMA therapy. In the specific cohorts from our group, an extrafine fixed-dose formulation was used and may have enhanced peripheral lung deposition, potentially contributing to improvement in small-airway physiology. However, these observations do not establish that particle size or distal deposition is responsible for remission, and comparative evidence demonstrating higher remission rates with extrafine versus non-extrafine triple therapy is currently lacking. Recent literature has further emphasized that LAMAs should not be regarded solely as bronchodilators in difficult-to-treat and severe asthma, but as components of triple therapy with potentially relevant effects on airway inflammation and remodelling-related pathways [31]. Subsequently, the concept evolved into steady quiet asthma, describing a durable biologic-free low-disease-activity state maintained for 12 months in approximately one-third of patients receiving optimized inhaled therapy alone, with baseline small-airway physiological indices predicting its achievement [16]. This severe-asthma-specific evidence derives from a small prospective cohort (n = 26) and should therefore be considered hypothesis-generating rather than evidence of a formulation-specific remission effect. At present, independent prospective confirmation of this proposed mechanistic link between extrafine therapy, small-airway stabilization, and clinical remission is lacking.

Preliminary, hypothesis-generating support for this concept comes from small mechanistic studies from our group showing lower exhaled acetylcholine concentrations and improved oscillometric indices in patients receiving extrafine triple therapy. Complementary evidence from a pilot study evaluated fractional exhaled nitric oxide measured at an expiratory flow of 350 mL/s (FeNO_350_). Unlike conventional FeNO measured at 50 mL/s, the higher expiratory flow reduces the relative contribution of central airway nitric oxide and may provide complementary information on more peripheral airway inflammation. In this pilot study, baseline FeNO_350_ was an independent predictor of subsequent clinical remission, although this measurement remains exploratory and requires validation in larger cohorts [32,33].

Taken together, the available evidence supports the hypothesis that quiet asthma may represent a biologically grounded phenotype characterized by sustained small-airway stabilization rather than an alternative definition of clinical remission. When this state is maintained over time and accompanied by the multidomain criteria required for remission—including control of exacerbations and symptoms, absence of oral corticosteroid dependence, and stable lung function—it may represent one potential pathway toward biologic-free clinical remission. This interpretation remains hypothesis-generating and requires prospective validation in larger, independently characterized cohorts [1,2,15,16,28,30,32,33].

## 7. Durability of Remission and Risk of Relapse

The achievement of biologic-free clinical remission has important clinical implications beyond symptom improvement. Sustained remission is associated with freedom from severe exacerbations and maintenance oral corticosteroids, fewer emergency visits and hospitalizations, improved quality of life, and reduced treatment burden. Even partial clinical remission may represent a meaningful therapeutic outcome when it substantially reduces exacerbation frequency and oral corticosteroid exposure [1,2,3,4,5,6,9,15,16,17,18].

However, remission should not be equated with cure. A population-based cohort of adults with spontaneous asthma remission demonstrated that residual biological abnormalities, including persistent bronchial hyperresponsiveness and specific cytokine patterns, predicted subsequent relapse and lung function decline despite apparent clinical quiescence [34]. These findings raise the possibility that patients achieving remission with optimized inhaled therapy may still harbor ongoing disease activity that is not clinically evident [34]. Accordingly, remission should be regarded as a sustained but potentially reversible disease state rather than permanent resolution. In patients who remain on optimized high-dose inhaled therapy, this state should therefore be interpreted as clinical remission on treatment rather than treatment-free remission. Treatment-free remission should be reserved for sustained remission after withdrawal of maintenance asthma therapy, whereas complete remission additionally implies resolution of underlying disease activity according to the definition applied. Continued multidomain follow-up—including assessment of symptoms, exacerbations, lung function, oral corticosteroid exposure and, where available, biological markers—remains essential to confirm the durability of remission and identify early relapse [1,2,3,4,5,13,34].

## 8. Clinical and Economic Implications

The clinical implications of achieving remission without biologic therapy extend beyond disease control and may also have relevant health-economic implications. Severe asthma accounts for a disproportionate share of healthcare expenditure because a relatively small subgroup of patients generates substantial use of emergency care, hospitalizations, chronic oral corticosteroids, work absenteeism, and treatment-related morbidity. Consequently, remission achieved during optimized inhaled therapy may plausibly reduce healthcare utilization and treatment burden in selected patients; however, direct evidence demonstrating a cost-effectiveness advantage over biologic treatment is currently lacking [35].

A major contributor to this burden is chronic oral corticosteroid exposure. In a Swedish observational cohort, regular oral corticosteroid use in asthma was associated with substantially greater healthcare resource utilization than no oral corticosteroid exposure, with inpatient care representing a major driver of expenditure [36]. Likewise, a systematic review demonstrated the substantial clinical and healthcare burden associated with systemic corticosteroid exposure [37]. Accordingly, potential health-economic benefits may include reduced corticosteroid-related morbidity and healthcare utilization and, in appropriately selected patients, avoidance of unnecessary treatment escalation. These consequences remain plausible rather than established economic benefits of biologic-free remission and require dedicated comparative health-economic analyses [38,39].

These potential economic advantages should, however, be interpreted within an appropriate clinical context. Pursuing inhaled-only remission in patients with persistent type 2 inflammation despite optimized inhaled therapy should not delay initiation of biologic treatment when clearly indicated. In such patients, postponing biologic therapy may allow exacerbations, cumulative corticosteroid exposure, hospitalizations, and irreversible loss of lung function to accrue, ultimately increasing both clinical burden and healthcare costs. Taken together, the available evidence supports a strategy of therapeutic optimization rather than therapeutic delay while biologic therapy should be introduced promptly when sustained multidomain remission is unlikely to be achieved with inhaled treatment alone [3,4,5,6,9,15,16,17,18,36,37].

## 9. Future Directions

The principal challenge is no longer to demonstrate that biologic-free remission can occur, but to identify the patients in whom it is achievable, define the mechanisms that sustain it, and establish its long-term durability [3,4,5,10,21]. Current evidence remains limited by the scarcity of dedicated longitudinal studies enrolling rigorously characterized severe asthma populations treated with optimized maximal inhaled therapy at GINA step 4 or 5 intensity without biologics [10,16,21,22,26,27,28,29].

Most available data derive from biologic registries, post hoc or retrospective analyses, and expert consensus frameworks, whereas inhaled-only cohorts notably include patients with significantly less severe disease and incomplete phenotyping [2,3,4,5,10,21,22,23,24,25,26,27,28,29]. Moreover, additional potential limitations are represented by the short observation period, frequently limited to 12 months, and the heterogeneity in the definition of remission across different studies [3,4,5,21].

Future prospective studies should therefore identify the clinical and biological characteristics that distinguish patients capable of achieving remission with inhaled therapy alone from those requiring biologic treatment, adopt harmonized multidomain remission definitions to facilitate comparisons across studies, and establish the long-term durability of remission together with predictors of relapse. Achieving these objectives will require comprehensive physiological and biological phenotyping, including spirometry, assessment of small-airway function, biomarker profiling, oral corticosteroid exposure, exacerbation history, patient-reported outcomes, quality of life, healthcare utilization, and health-economic measures. Particular emphasis should be placed on measures of distal airway disease, including oscillometric indices and peripheral airway inflammatory biomarkers, which may improve the identification of patients most likely to benefit from optimized inhaled therapy.

Importantly, ongoing prospective trials are beginning to directly address the optimal timing of biologic therapy relative to escalation of inhaled treatment. AIM4: Next Step (NCT06572228) compares earlier introduction of dupilumab added to medium-dose ICS/LABA with escalation to high-dose ICS/LABA in patients with uncontrolled type 2 asthma [39], whereas BRISOTE (NCT06750289) compares benralizumab added to medium-dose ICS/LABA with conventional escalation to high-dose ICS/LABA in uncontrolled eosinophilic asthma [40]. These studies, including assessment of multidomain clinical outcomes and, in BRISOTE, a four-component clinical remission endpoint, may provide important prospective evidence on whether earlier biologic intervention offers advantages over further intensification of inhaled therapy.

Future research should also clarify the biological mechanisms potentially contributing to remission achieved during optimized inhaled therapy, including the contribution of pharmacological synergy, improved peripheral drug deposition, and restoration of small-airway homeostasis. Whether this synergistic mechanism proposed by Rogliani and colleagues translates into the achievement of multidomain clinical remission, rather than isolated physiological improvements, remains to be established [14,19]. Addressing this question will be essential to determine whether biologic-free remission represents an exceptional clinical outcome or a reproducible therapeutic pathway for carefully selected patients with severe asthma.

## 10. Conclusions

Overall, the available evidence indicates that clinical remission with optimized inhaled therapy may be achievable in selected patients across difficult-to-treat and severe asthma populations, although the evidence specifically derived from rigorously defined severe asthma remains more limited. In patients with persistent Type 2-high severe asthma who are eligible for biologic treatment, biologic therapies remain highly effective remission-oriented options. However, within the therapeutic pathway preceding escalation to biologic treatment, the available evidence indicates that sustained multidomain clinical remission may still be achieved during rigorous optimization of inhaled triple therapy in a clinically relevant subset of patients. This window is particularly important in patients whose disease remains responsive to conventional treatment, in whom modifiable factors and treatable traits can be corrected and disease stability can be achieved without immediate biologic escalation. Thus, the role of optimized inhaled triple therapy should be viewed as complementary to, rather than competitive with, biologic therapy: biologics remain the preferred option when severe Type 2-driven disease persists despite full optimization, whereas selected patients may achieve remission before escalation is required [3,4,5,6,9,21]. Importantly, some inhaled-therapy-only cohorts, including the SANI subgroup [9], represented patients with less severe disease than those receiving or eligible for biologic treatment; therefore, their remission rates should not be directly extrapolated to the overall severe-asthma population. Importantly, achievement of remission following correction of previously unrecognized modifiable factors may retrospectively identify difficult-to-treat rather than severe asthma. By contrast, remission occurring after further optimization with triple therapy in patients who had already fulfilled severe-asthma criteria on optimized high-dose ICS/LABA can reasonably be regarded as on-treatment remission of severe asthma. In this broader perspective, remission represents part of an evolving disease-modification and treat-to-target framework rather than an outcome restricted to any single therapeutic class [11].

Within this framework, quiet asthma provides a useful conceptual model linking small-airway stabilization with biologic-free disease quiescence, while the pharmacological synergy of optimized triple therapy offers a plausible biological explanation for the multidomain improvements required to achieve remission. Together, these concepts support the view that optimized inhaled therapy should be considered an established component of asthma management and a potential remission-oriented strategy in appropriately selected patients, including those in whom clinical remission may be achieved without biologic therapy.

The next challenge is no longer to establish whether biologic-free clinical remission is possible, but to identify the patients most likely to achieve it, define the mechanisms that sustain it, and prospectively validate remission-oriented inhaled treatment strategies. Achieving these goals will determine the role of biologic-free remission within precision asthma management and identify the difficult-to-treat and severe asthma populations in whom this approach can be most appropriately applied.

## Figures and Tables

**Table 1:** Key longitudinal studies of biologic-free clinical remission with optimized inhaled therapy.

Article (Reference)	Study	Design	n	Study Population	Treatment	Remission Criteria *	Main Result	Clinical Message
Gessner et al. [26]	TriMaximize	Prospective real-world	1445	Moderate-to-severe asthma receiving BDP/FOR/G in real-world practice	BDP/FOR/G	No exacerbations + no SCS + ACT ≥ 20 + stable FEV_1_	4-component remission: 39.5% (Year 1); 47.9% (Years 2–3)	Large prospective real-world cohort
Noorduyn et al. [29]	PERFORM	Pragmatic RCT	1236	Uncontrolled, predominantly infrequently exacerbating asthma; not specifically severe asthma	FF/UMEC/VI vs. ICS/LABA	No exacerbations + no OCS + ACQ < 1.5 + FEV_1_ improvement	29.7% vs. 17.0% remission	Pragmatic randomized evidence in uncontrolled asthma
Canonica et al. [27]	TRIMARAN/TRIGGER	Post hoc Phase III RCTs	2291	Uncontrolled asthma with ≥1 previous exacerbation despite medium-/high-dose ICS/LABA	BDP/FOR/G vs. BDP/FOR	No exacerbations + no OCS + ACQ-5 < 1.5 + stable FEV_1_	29.5% vs. 22.7% remission	Post hoc remission analysis of Phase III trials
Oppenheimer et al. [25]	CAPTAIN	Post hoc Phase III RCT	2436	Uncontrolled moderate-to-severe asthma despite ICS/LABA	FF/UMEC/VI vs. FF/VI	No exacerbations + no OCS + ACQ control + stable/improved FEV_1_	Higher remission with triple therapy	Post hoc remission analysis in moderate-to-severe asthma
Canonica et al. [9]	SANI Registry	National registry	58 inhaled-only	Patients managed within a severe-asthma registry; inhaled-only subgroup	Optimized inhaled therapy	No OCS + no exacerbations + symptom control + stable FEV_1_	34.5% complete remission	Real-world evidence from a severe-asthma registry subgroup
Quaranta et al. [16]	Steady Quiet Asthma	Prospective real-world cohort	26	Severe asthma with small-airway dysfunction, prospectively treated with extrafine SITT	Extrafine BDP/FOR/G	Sustained control + no exacerbations/OCS + stable lung & small-airway function	34.6% biologic-free at 12 months	Hypothesis-generating severe-asthma cohort
Kumar et al. [28]	ATLANTIS	Post hoc longitudinal exploratory analysis of a multinational prospective cohort	684	Adults with asthma across a broad severity spectrum; not a dedicated severe-asthma cohort	Background asthma therapy; observational analysis rather than treatment randomization	3C: ACQ-6 < 1.5 + no maintenance OCS + no exacerbations; 4C: 3C + <10% decline in pre-BD FEV_1_% predicted	48% achieved 3C remission; 45% achieved 4C remission	Less severe small-airway dysfunction and greater functional reserve were associated with remission

* Criteria abbreviated for readability; original definitions differed slightly among studies. Remission criteria are shown in abbreviated, study-specific form. All composite endpoints required multidomain stability, typically including absence of severe exacerbations, no maintenance or frequent oral/systemic corticosteroid use, well-controlled symptoms (ACT or ACQ thresholds), and stable or improved lung function (FEV_1_). Exact definitions and thresholds are reported in the original publications. Terminology is reported according to the definition used in each original study; therefore, ‘clinical remission’, ‘complete clinical remission’, and other composite remission endpoints are not assumed to be interchangeable across studies. Abbreviations: ICS = inhaled corticosteroid; LABA = long-acting β_2_-agonist; LAMA = long-acting muscarinic antagonist; BDP = beclometasone dipropionate; FOR = formoterol; FF = fluticasone furoate; G = glycopyrronium; FF/UMEC/VI = fluticasone furoate/umeclidinium/vilanterol; SCS/OCS = systemic/oral corticosteroids; ACT = Asthma Control Test; ACQ = Asthma Control Questionnaire; FEV_1_ = forced expiratory volume in 1 s; RCT = randomized controlled trial; SANI = Severe Asthma Network Italy.

## Data Availability

No new data were created or analyzed in this study. Data sharing is not applicable to this article.
